# New and Innovative Treatments for Neovascular Age-Related Macular Degeneration (nAMD)

**DOI:** 10.3390/jcm10112436

**Published:** 2021-05-30

**Authors:** Prem Patel, Veeral Sheth

**Affiliations:** 1 Department of Ophthalmology, University of Texas Southwestern Medical Center, Dallas, TX 75390, USA; prem.patel@utsouthwestern.edu; 2 University Retina and Macula Associates, Oak Forest, IL 60452, USA

**Keywords:** emerging treatment, neovascular age-related macular degeneration (nAMD), Vascular Endothelial Growth Factor (VEGF)

## Abstract

Age-related macular degeneration (AMD) is one of the most common causes of vision loss. Advanced forms of AMD are seen in primarily two types—neovascular AMD (nAMD) with the presence of choroid neovascularization and non-neovascular AMD (nnAMD) with geographic atrophy. Neovascular AMD is characterized by choroidal neovascularization (CNV), which leads to a cascade of complications, including exudation, leakage, and ultimately fibrosis with photoreceptor loss. Inhibition of VEGF represents the current standard of care. However, there is a tremendous gap between the outcomes in randomized clinical trials and real-world settings. New agents for nAMD might offer the potential to improve treatment outcomes and reduce treatment of frequent intravitreal injections. We summarize all the newer molecules, their pivotal clinical trial results, and their unique mechanisms of action; these include longer-acting agents, combination strategies, sustained release, and genetic therapies.

## 1. Introduction

Age-related macular degeneration (AMD) is a leading cause of degenerative vision loss in elder individuals [1,2,3]. Due to an aging population, the global prevalence of AMD is projected to rise from 170 to 288 million by the year 2040 [4]. AMD may be classified as early, intermediate, and advanced types based on severity [5,6]. In early AMD, multiple small- and medium-sized drusen lipids deposit under the retina, or there are mild pigmentation abnormalities of the retinal pigment epithelium (RPE) in at least one eye. Intermediate AMD is characterized by at least one large druse, retinal pigment abnormalities, or geographic atrophy of the RPE that does not involve the center of the fovea. Lastly, advanced AMD is vision threatening and is seen in primarily two types—neovascular AMD (nAMD) and non-neovascular AMD (nnAMD) with geographic atrophy.

“Wet” or neovascular AMD (nAMD) is defined by choroidal neovascularization (CNV) that causes bleeding, fluid accumulation, and fibrosis of the macula [7]. While CNV only affects 10–15% of patients diagnosed with AMD, it accounts for 90% of severe vision loss caused by AMD [8,9]. Macular photocoagulation has been historically used to limit damage from choroidal lesions [10]. However, the past 15 years have experienced a paradigm shift in the treatment of nAMD. Intravitreal antivascular epithelial growth factor (VEGF) agents—bevacizumab, ranibizumab, and aflibercept—now represent the standard of care. Additionally, a fourth intravitreal drug, brolucizumab, was approved by the FDA in the last quarter of 2019. A large body of evidence from randomized clinical trials has helped to guide clinicians to use these agents with great success.

However, despite their proven efficacy, anti-VEGF agents still face issues. Firstly, there is a high treatment burden due to their short duration of action. Patients may require monthly injections over many years of treatment. Furthermore, long-term administration of intravitreal anti-VEGF injections is not ideal. Studies reflect worse visual acuity outcomes in the real world than those achieved in clinical trials [11,12]. This may be explained by challenges with compliance to regular injections, resulting in a large share of real-world patients being undertreated. Additionally, anatomic features, such as the development of fibrosis, may affect this outcome. In the CATT study, 25% of patients on aggressive anti-VEGF therapy developed some degree of fibrosis at 2 years [13]. Furthermore, there was an increased risk of developing retinal scarring and geographic atrophy in nAMD patients 2 to 5 years after initiating treatment [14]. Complications such as vitreous and subconjunctival hemorrhage, fluid accumulation under the fovea, increased intraocular pressure, endophthalmitis, and ocular inflammation have also been described [15,16,17,18].

There is clearly an unmet need for more durable and longer-acting treatment against nAMD. Several promising agents are in development, which improve upon current anti-VEGF therapy, exploit novel pathways, use innovative delivery systems, or offer combination therapy. This review aims to summarize these emerging therapies, their mechanisms of action, and their pivotal clinical trial results (Table 1).

## 2. Methods of Literature Search

The literature search was conducted by searching PubMed and Google Scholar, along with sources cited from companies’ websites. The latter allowed us to locate findings that were presented at recent conferences. Only articles in the English language were included. The search was conducted up to the end of April 2021.

## 3. Pathophysiological Aspects of Current and Future Therapy

The pathophysiology of AMD is multifactorial and complex. In addition to strong age dependence, there are a variety of metabolic, functional, genetic, and environmental factors at play [19,20,21,22,23,24,25,26]. At least four key processes contribute to disease: lipofuscinogenesis, drusogenesis, neovascularization, and local inflammation [27].

With aging, several metabolites accumulate within the retina, leading to elevated levels of the age-related pigment, lipofuscin [27]. Lipofuscin is the product of incomplete metabolism of external segments of photoreceptors by phagolysosomes. Elevated concentrations of this pigment have been associated with cell damage and oxidative stress. These toxic effects impair the RPE, which is responsible for the maintenance of photoreceptor cells and is involved in the recycling of visual pigments and daily phagocytosis of constantly shed photoreceptor outer segments. Additionally, A2E (breakdown product of lipofuscin) has been found to activate the complement system, further contributing to pathogenesis [21].

Another component of AMD pathogenesis is the development of lipid deposits called drusen, which may be “soft” or “hard” depending on size and shape. Drusen are composed of similar protein components to the plaques found in Alzheimer’s disease [20]. Soft drusen appear as large, pale-colored, dome-shaped elevations that can resemble localized serous RPE detachments. In contrast, large drusen are usually a sign of diffuse thickening of Bruch’s membrane with basal linear deposit. Studies suggest that local inflammation and activation of the complement cascade actively contribute to drusogenesis, photoreceptor degeneration, and Bruch’s membrane disruption.

Lastly, choroidal neovascularization (CNV) characterizes pathology for which nAMD gets its “wet” name. CNV leads to uncontrolled growth of leaky blood vessels under the macula in a variety of exudative eye conditions, such as AMD and diabetic retinopathy. This process may be mediated, in part, by local inflammation and immune reactions [21]. Neutrophils, macrophages, mast cells, and activated microglia can release an array of proangiogenic factors, including VEGF [28]. The VEGF family of proteins regulate vascular permeability in the retina and are the target of current therapy.

Among these proteins, VEGF-A is the principal driver of CNV, binding to the extracellular ligand-binding domains of two tyrosine kinase receptors (VEGFR-1 and VEGFR-2). This cascade leads to the activation of genes for angiogenesis and vascular permeability. However, aside from VEGF-A, there has been recent therapeutic interest in targeting VEGF-C and VEGF-D. These isoforms have been shown to be increased in response to inhibition of VEGF-A [29,30,31,32]. Selective blockade of VEGF-A may trigger compensatory upregulation of other members of the VEGF family. Therefore, avenues to suppress this mechanism of resistance are under active exploration.

**Figure 1:**
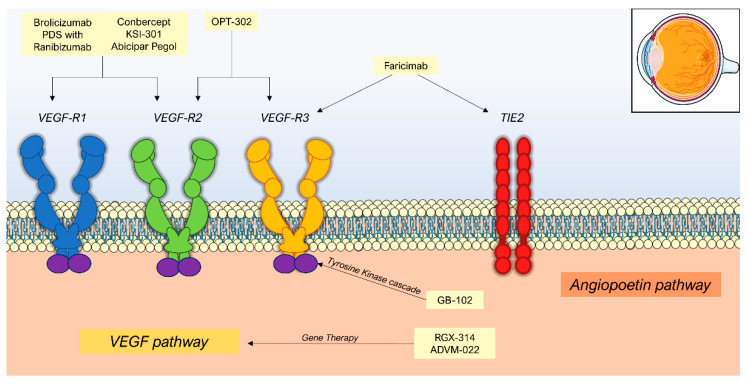
Mechanisms of action of Neovascular age-related macular degeneration therapies in development.

Additionally, angiogenesis receives a contribution from alternative pathways, such as the Ang-Tie 2 axis. In this pathway, angiopoietin-1 and angiopoietin-2 are key cytokines that interact with transmembrane receptor tyrosine kinase (Tie-2) [33]. In healthy states, Tie-2 is bound by angiopoietin-1, which is a protective factor, promoting vascular stability, pericyte recruitment, and the inhibition of vascular permeability factors [34]. However, in angiogenic states, the competitive inhibitor angiopoietin-2 is upregulated, displacing Ang-1, and causing endothelial destabilization, inflammation, and breakdown of the blood–retina barrier [35]. Combination therapies that target these non-VEGF angiogenic factors may provide additional benefit over current standard of care. However, this is yet to be determined. The agents discussed in this review are organized according to the mechanism of action in Figure 1.

## 4. Emerging Neovascular AMD Therapies

### 4.1. Faricimab

The current first-line therapy for nAMD exclusively targets VEGF, creating an unmet need for anti-VEGF subresponsive patients. Faricimab aims to fill this void by exploiting the alternative Ang-2/Tie-2 pathway [33]. Faricimab is the first bispecific monoclonal antibody designed for intraocular use [34]. With two arms, the antibody independently binds and neutralizes both VEGF-A and angiopoetin-2 (Ang-2), which together synergistically promote vascular stability.

TENAYA and LUCERNE were Phase 3, multicenter, randomized studies to evaluate the efficacy and safety of faricimab in patients with nAMD [36,37]. Patients were randomized to receive faricimab 6 mg up to every 16 weeks or aflibercept 2 mg every 8 weeks after three initial loading doses. With the faricimab arm, they had four initial loading doses with disease activity assessments at Weeks 20 and 24 based on best-corrected visual acuity (BCVA) and central subfield thickness (CST) criteria, along with the investigator’s evaluation. At Week 20, if there was active disease, those patients continued on at every 8 weeks. At Week 24, if there was active disease, those patients continued on for every 12 weeks. If there was no disease activity, those patients maintained every 16-week interval with faricimab. The primary endpoint was change in BCVA from baseline averaged over three visits, Weeks 40, 44, and 48. Results showed meaningful and comparable reductions in CST from baseline through Week 48 with faricimab up to every 16 weeks and aflibercept every 8 weeks. The median number of injections for the faricimab arm was six vs. eight in aflibercept. The initial BCVA gains were sustained, with a majority of patients in the faricimab arm up to every 16 weeks. Faricimab showed excellent durability with 45% of patients on every 16-week and almost 80% of patients on every 12-week dosing by Week 48. Concerning safety, the rates of intraocular inflammation in these studies were low. Intraocular inflammation was reported on average in 2% and 1.2% of patients for faricimab and aflibercept, respectively. There were no cases of vasculitis.

TENAYA and LUCERNE are 2-year studies; the long-term extension study, AVONELLE-X, will generate 4-year long-term data. These data on faricimab offer hope of exploiting additional mechanisms beyond VEGF. With much still being elucidated about the role of angiopoietin in vessel stabilization, it will be interesting to study if faricimab provides additional benefits from having a unique mechanism of action.

### 4.2. Port Delivery System (PDS)

The success of traditional anti-VEGF therapies raised the question of whether a longer-acting ranibizumab could be delivered via an implantable reservoir. Thus, the Port Delivery System (PDS) was developed, allowing for the continuous release of ranibizumab into the vitreous via passive diffusion [38]. PDS is intended to reduce the frequency of intravitreal injections and potentially allow patients with nAMD to go several months before needing a refill of the implant. The device is a self-sealing eye implant that requires surgical implantation and can be refilled in the office via injection through the conjunctiva. Currently, the PDS holds 20 µL of a customized formulation of ranibizumab (100 mg/mL). This dosage was found to be the most effective dose from the Phase 2 LADDER trial in wet AMD, looking at visual and anatomic success [39].

Results of the Phase 3 ARCHWAY trial showed that PDS at every 24 weeks was noninferior and equivalent to monthly ranibizumab at its primary endpoint at Week 40 [40]. As expected, there was a transient postsurgical drop in vision in the PDS arm that recovered by Week 40. By Week 72, patients in the PDS arm had two refill exchanges at Weeks 24 and 48, with vision and anatomic outcomes comparable with monthly ranibizumab. There was equivalent vision and controlled retinal thickness, and PDS patients required five times fewer treatments over a mean duration of 78 weeks.

The idea of a surgically implanted VEGF depot is intriguing but does carry potential risks. VEGF has been found to be a key neurotrophic factor involved in the maintenance of retinal vasculature [41]. Potent, long-term inhibition may be disruptive to the health of neurovascular cells. There is evidence of increased risk of geographic atrophy in patients treated monthly as opposed to patients treated pro re nata [42,43]. With any of the extended durability anti-VEGF treatments in development, including PDS, little is known about the adverse events associated with the prolonged antagonism of VEGF. Additional considerations include risks from the surgical procedure itself and the possibility of endophthalmitis or vitreous hemorrhage. These risks are still being evaluated by the FDA for consideration for use. Nonetheless, the promising results from ARCHWAY are an excellent step towards increased longevity of nAMD therapy.

### 4.3. Abicipar Pegol

New to the world of protein therapeutics, designed ankyrin repeat protein (DARPin) molecules are small, single-domain proteins that can selectively bind to a target protein with high affinity and specificity [44,45]. These molecules are highly stable, providing advantages over currently available antibodies or antibody fragments. At present, abicipar pegol is a DARPin developed for use against nAMD [46]. Abicipar binds all isoforms of VEGF-A with excellent tissue penetration. Furthermore, it has a longer intraocular half-life compared with ranibizumab (>13 days vs. 7.2 days) [47].

Following encouraging results from the Phase 2 REACH study [48], two identical global Phase 3 studies were conducted (CEDAR and SEQUOIA) [49]. Participants with nAMD were divided into three arms: three monthly abicipar 2 mg injections followed by an injection every 8 weeks, two monthly abicipar 2 mg injections followed by an injection after 8 weeks and every 12 weeks thereafter, and monthly ranibizumab injections. Results showed mean change in BCVA during Year 2 was similar when compared to Year 1 across all treatment arms. Precisely 93% of patients in the 8-week abicipar group, 90% of patients in the 12-week abicipar group, and 94% of patients in the 4-week ranibizumab group achieved stable vision. Only four intravitreal injections of abicipar were required to maintain the outcomes, as compared to monthly intravitreal ranibizumab injections. Overall, abicipar demonstrated non-inferiority compared with ranibizumab, meeting its primary endpoint.

However, roughly 15% of abicipar-treated eyes experienced intraocular inflammation (IOI). In efforts to reduce this adverse effect, the manufacturing process has since been modified. The MAPLE study, a 28-week safety evaluation, was performed to determine the rates of adverse events in 128 patients after the manufacturing process was changed. The data showed a reduced intraocular inflammation rate of 8.9%, and only 1.6% of these cases were deemed moderately severe or severe [50]. The improvement in the rate of adverse effects is because reformulation is a step in the right direction; however, additional research is required to validate the efficacy and extended duration of abicipar.

### 4.4. Brolucizumab

At a size of ~26 kDa, the humanized single-chain antibody fragment brolucizumab may provide enhanced tissue penetration, clearance, and drug delivery characteristics compared to more traditional anti-VEGF agents [51]. By comparison, ranibizumab and aflibercept have molecular weights of 48 and 115 kDa, respectively [52]. The molar dose of brolucizumab is 11.2 to 13.3 times higher than that of aflibercept, permitting greater drug concentrations and therefore longer duration.

The safety and efficacy of brolucizumab were compared to aflibercept in two Phase 3 trials, HAWK and HARRIER [53]. The primary endpoint in both studies was noninferiority to aflibercept in mean change in BCVA from baseline to Week 48. In HAWK, patients were randomized to intravitreal brolucizumab 3 mg, brolucizumab 6 mg, or aflibercept 2 mg. HARRIER randomized patients to brolucizumab 6 mg or aflibercept 2 mg. Brolucizumab was noninferior to aflibercept in the primary outcomes in both studies. In the superiority analysis of HAWK at Week 16, the incidence of disease activity was significantly lower with brolucizumab 6 mg compared with aflibercept (24.0% vs. 34.5%). Intraretinal fluid/SRF was present in fewer brolucizumab-treated eyes versus aflibercept-treated eyes at Week 16 in both trials. Rates of ocular and nonocular AEs were similar with brolucizumab and aflibercept.

Despite the efficacy of brolucizumab for nAMD, and its superior pharmacokinetics, many retina specialists are concerned about the risk of occlusive vasculitis and blindness with the drug. The rate of uveitis was 2.2% with brolucizumab 6 mg and 0.3% with aflibercept in HAWK, and <1% with both drugs in HARRIER. The incidence of iritis was 2.2% with brolucizumab 6 mg and 0% with aflibercept in HAWK, and <1% with both drugs in HARRIER. While the FDA has recently given approval towards brolucizumab use, it is unclear whether these adverse events will outweigh the potential benefits.

### 4.5. KSI-301

Mechanically, KS-301 resembles the classic anti-VEGF agents; however, it is based on a 950 kDa antibody biopolymer conjugate (ABC) platform that is engineered specifically for increased durability [54]. Preclinical pharmacokinetic studies have demonstrated KSI-301’s extended ocular half-life of 10–12 days. In a Phase 1b study, patients received three loading doses at Weeks 0, 4, and 8. There was a durability assessment from Weeks 12 to 72 with an extension study from weeks 76 to 148. The efficacy of KSI-301 was determined by change from baseline to Week 52 in mean BCVA and optical coherence tomography (OCT) thickness. There was an observed mean 5.7-letter improvement to 69.7 ETDRS eye chart letters (~20/40 Snellen) at Year 1 [55]. Additionally, thickness had decreased by 105 microns. Patients received three loading doses, followed by an average of two individualized doses thereafter, resulting in a total of five mean injections in Year 1.

There was an excellent safety profile for KSI-301. Most adverse effects were assessed as mild and consistent with the profile of intravitreal (IVT) anti-VEGF injections. To date, 43 serious AEs (SAEs) have been reported in 24 subjects; however, none were drug related. Additionally, three ocular SAEs in the study eye not drug related were all resolved. Only two AEs of IOI (2/710, 0.28%) were noted, both traced to 1+ vitreous cell with complete resolution.

### 4.6. Conbercept

Conbercept is a 141 kDa engineered fusion protein that, like aflibercept, acts as receptor decoy against VEGF [56]. However, conbercept has higher binding affinity and contains an additional fourth binding domain of VEGFR2. This design is hypothesized to provide increased stability of the receptor-ligand complex and extend the half-life of conbercept [56,57,58,59]. Two global Phase 3 trials for nAMD were initiated: PANDA-1 and PANDA-2. Each trial is evaluating 1140 patients randomized to conbercept, 0.5 or 1 mg, or aflibercept 2 mg, with primary efficacy analysis at 36 weeks. In PANDA-1, patients received three loading doses through Week 8, then continued with dosing every 8 weeks through Week 92. In PANDA-2, dosing was pro re nata after Week 40, with the 0.5 mg conbercept and aflibercept groups on the same regimen as PANDA-1 up until that point, after which the conbercept 1 mg arm shifted to 12-week dosing after 8 weeks and moved onto PRN at Week 40. The PANDA trials recently reached a milestone by completing 36-week primary endpoint visits of enrolled patients in December 2020.

### 4.7. OPT-302

With traditional VEGF blockade, current therapies target VEGF-A, which is considered to be the most pathologic isoform. However, in this process, other VEGF isoforms are upregulated [29,30,31,32]. OPT-302 is a novel “trap” molecule that binds and neutralizes the activity of VEGF-C/-D, blocking their activation of receptors VEGFR2 and VEGFR-3 [60]. There is hope that combining OPT-302 with currently available anti-VEGF-A may address mechanisms of resistance associated with existing therapies. Two concurrent global Phase 3 trials known as Study of OPT-302 in combination with Ranibizumab (ShORe) and Combination OPT-302 with Aflibercept Study (COAST) have begun [61]. These trials build upon the successful Phase 2b nAMD clinical trial while additionally evaluating the administration of OPT-302 in combination with ranibizumab and aflibercept over a longer treatment period and in a greater number of patients. ShORe and COAST will enroll approximately 990 treatment-naive patients each and assess the efficacy and safety of intravitreal 2.0 mg OPT-302 in combination with 0.5 mg ranibizumab or 2.0 mg aflibercept, compared to ranibizumab or aflibercept monotherapy, respectively. The primary endpoint of both studies is the mean change in BCVA from baseline to Week 52 for OPT-302 combination therapy compared to anti-VEGF-A monotherapy.

### 4.8. GB-102

Like PDS, GB-102 is another sustained-release anti-VEGF delivery system [62]. However, GB-102 is formulated as an intravitreal formulation of sunitinib malate-containing, biodegradable microparticles. The controlled microparticle release is intended for biannual injection to maintain comparable visual acuity and central subfield thickness outcomes. The ADAGIO Phase 1/2a study consisted of patients with nAMD who received four escalating dose cohorts of eight patients, each receiving a single dose of either 0.25, 0.5, 1, or 2 mg of GB-102 [63]. Precisely 88% of the patients at 3 months and 68% of the patients at 6 months were maintained on a single dose of GB-102. Positive outcomes were observed for up to 8 months. CST was significantly reduced at all months compared with pretreatment. However, one concern that emerged was the nonaggregation of the drug once in the vitreous cavity, resulting in particle dispersion. Nine of thirty-two subjects experienced related symptoms, including eye pain, photophobia, and blurriness [64]. A new manufacturing process was developed to eliminate the microparticle dispersion and incomplete aggregation, which was used for future trials.

Phase 2b ALTISSIMO was initiated to further evaluate GB-102 for CNV lesions in previously treated nAMD patients [65]. The study consists of three cohorts: 1 mg of GB-102, 2 mg of GB-102, or 2 mg of aflibercept at baseline. The GB-102 cohorts will then receive their same initial dose every 6 months, whereas the latter control group will continue to receive aflibercept 2 mg every 2 months. The primary outcome is the proportion of treated subjects remaining rescue free through Month 10. While the efficacy of GB-102 is still being determined, it provides an immediate bridge towards longer-lasting therapies further down the pipeline. GB-102 has by far the longest time needed between treatments, and the development of GB-103, which aims for once-a-year dosing, has already begun

### 4.9. RGX-314

Gene therapy has shown promise for the treatment of inherited retinal diseases, and recently there has been a push for gene therapy solutions for nAMD. RGX-314 is a vector designed to bind and neutralize VEGF in a manner similar to ranibizumab [66]. RGX-314 utilizes adeno-associated virus serotype 8 (AAV8) as its vector, with research suggesting that AAV vectors provide long-term transgene expression [67]. The gene therapy vector is preferentially taken up by retinal cells, leading to high levels of production of the monoclonal antibody fragment. The company is advancing two separate routes of ocular administration of RGX-314: a one-time subretinal administration during vitrectomy; and in-office suprachoroidal delivery. The hope is that the long-standing and stable production of the anti-VEGF therapeutic protein could reduce the need for frequent intravitreal injections.

ATMOSPHERE is the first of two planned pivotal trials for the evaluation of subretinal delivery of RGX-314 in patients who have received prior treatment for nAMD [68,69]. Patients underwent vitrectomy and were delivered subretinal RGX-314 across five dose cohorts (3 *×* 10^9^ genome copies (GC)/eye, 1 *×* 10^10^ GC/eye, 6 *×* 10^10^ GC/eye, 1.6 × 10^11^ GC/eye, 2.5 *×* 10^11^ GC/eye). RGX-314 continued to be generally well tolerated across all cohorts, with 20 serious adverse events (SAEs) reported in 13 patients, including 1 possibly drug-related SAE of a significant decrease in vision in Cohort 5. The most common nonserious adverse events in the eye were generally assessed as mild (87%). These included postoperative conjunctival hemorrhage (69% of patients), postoperative inflammation (36% of patients), eye irritation (17% of patients), eye pain (17% of patients), and postoperative visual acuity reduction (17% of patients). In 67% of patients across all cohorts, and in 83% of patients in Cohorts 3 through 5, retinal pigmentary changes were observed on imaging, the majority of which were in the peripheral inferior retina. Retinal hemorrhage was observed in 26% of patients and is an anticipated event in patients with severe wet AMD. There have been no reports of clinically determined immune responses, drug-related ocular inflammation, or postsurgical inflammation beyond what is expected following routine vitrectomy. In the two higher dose cohorts (four and five), patients at 1.5 years after treatment demonstrated stable visual acuity with a mean BCVA change of +1 letters and −1 letters from baseline, respectively, as well as decreased CRT, with a mean change of −46 and −93 µm, respectively. In Cohort 4, 4 out of 12 (33%) patients have received no anti-VEGF injections after 6 months following RGX-314 administration and demonstrated a mean BCVA change from baseline of +2 letters at 1.5 years. Eight out of eleven (73%) patients have received no anti-VEGF injections after 6 months following RGX-314 administration and demonstrated a mean BCVA change from baseline of −2 letters at 1.5 years. These data show a meaningful reduction in anti-VEGF treatment burden in both Cohorts 4 and 5. With the positive results of ATMOSPHERE, and the pending results of suprachoroidal RGX-314 delivery in AAVIATE, there is much promise for gene therapy in nAMD treatment.

### 4.10. ADVM-022

ADVM-022 is another gene therapy that aims to provide sustained anti-VEGF expression from the retina. In the OPTIC trial, the primary objective was to assess the safety and tolerability of a single IVT injection of ADVM-022. Secondary objectives were to evaluate BCVA and anatomy using spectral-domain OCT (SD-OCT), and to assess the need for rescue therapy [69]. All patients received aflibercept injection 1 to 2 weeks prior to dosing of ADVM-022. There was a 24-week safety and efficacy assessment. Again, the same was done at Week 52 with a follow-up at 104 weeks. Patients received oral steroid prophylaxis in Cohorts 1 and 2 and steroid eyedrop prophylaxis in Cohorts 3 and 4.

Overall, ADVM-022 continues to be well tolerated with a favorable safety profile at both high and low doses. It showed robust and sustained efficacy in both high and low doses. There was excellent durability out to 92 weeks from a single IVT injection with 0 supplemental injections in Cohort 1. There was robust aqueous anti-VEGF protein expression observed at 18 months in Cohort 1. This study showed a substantial reduction in annualized injection frequency following ADVM-022. Most patients did not require any supplemental injection in OPTIC. Patients completing 2 years in OPTIC are being enrolled into an extension trial to be followed for up to 5 years. Two global Phase 3 (PIVOTAL-a and PIVOTAL-b) trials are targeted to initiate in the fourth quarter of 2021.

Gene therapy differs from other extended durability therapies as no hardware is implanted in the eye. This may circumvent potential complications such as conjunctival erosion. Moreover, there is tremendous value in the potential role of gene therapy in preventing chronic exudative eye conditions such as nAMD, as many researchers believe that early intervention is valuable in limiting the progression of nAMD. While one or two intravitreal injections are generally tolerable, ongoing treatment with no definite cessation for patients who are asymptomatic can often be untenable for them. So, there is much excitement about the possibility of a one-time treatment with sustained intraocular VEGF suppression that could slow the course of nAMD. However, a potential disadvantage to gene therapy is the inability to turn it off. The consequences of long-term VEGF blockade are still being elucidated.

## 5. Conclusions

While anti-VEGF agents have revolutionized our treatment of nAMD, the field continues to evolve in the hope of providing better options for our patients. As discussed, numerous novel molecular targets may allow us to improve upon the clinical outcomes achieved by the VEGF blockade. Beyond VEGF, there are several trials underway investigating alternative factors in retinal and choroidal angiogenesis, such as PDGF, FGF, and EGF. Furthermore, research towards longer-acting pharmaceuticals might yield good results with fewer treatments, helping to improve compliance, possibly allowing us to treat more patients.

## Figures and Tables

**Table 1:** Currently available and experimental treatments for neovascular age-related macular degeneration (nAMD).

Drug	Mechanism of Action	Company	Relevant Studies	Phase of Study
Faricimab	Angiopoetin-2 and VEGF-A antibody	Genentech	TENAYA, LUCERNE, AVONELLE-X	3
Port Delivery System (PDS) with Ranibizumab	Surgically implanted reservoir with anti-VEGF	Genentech/Roche	ARCHWAY	3
Abicipar Pegol	Anti-VEGF DARPin	Allergan	CEDAR, SEQUOIA	3
Brolucizumab	Single-chain anti-VEGF antibody fragment	Novartis	HAWK, HARRIER	3
KSI-301	Antibody biopolymer conjugate	Kodiak Sciences	DAZZLE	1b
Conbercept	Recombinant VEGF receptor antibody	Chengdu Kanghong Biotech Company	PANDA-1, PANDA-2	3
OPT-302	VEGF-C and VEGF-D blockade	Molecular Partners	ShORe, COAST	3
GB-102	Depot formulation of sunitinib malate	Graybug Vision	ADAGIO, ALTISSIMO	1/2a, 2b
RGX-314	Gene therapy	REGENXBIO	ATMOSPHERE, AAVIATE	2b/3
ADVM-022	Gene therapy	Advernum Biotechnologies	OPTIC	1

## Data Availability

No new data were created or analyzed in this study. Data sharing is not applicable to this article.
